# Rational engineering of a virulence gene from *Mycobacterium tuberculosis* facilitates proteomic analysis of a natural protein N-terminus

**DOI:** 10.1038/srep33265

**Published:** 2016-09-14

**Authors:** Cristal Reyna, Felix Mba Medie, Matthew M. Champion, Patricia A. Champion

**Affiliations:** 1 Department of Biological Sciences, Eck Institute for Global Health, Center for Rare and Neglected Diseases, University of Notre Dame, Notre Dame IN, USA; 2 Department of Chemistry and Biochemistry, Eck Institute for Global Health, Center for Rare and Neglected Diseases, University of Notre Dame, Notre Dame IN, USA

## Abstract

Mass spectrometry (MS) for the detection of proteins is an indispensable tool for evaluating the biological processes of the proteome. Proteomics frequently requires proteolysis of proteins into peptide fragments. Proteins can be refractory to ideal proteolysis at the sequence level rendering them difficult to analyze by routine proteomics methods. EsxA (ESAT-6, Early Secreted Antigen, 6kDa) is a major virulence determinant of *Mycobacterium tuberculosis,* the cause of human tuberculosis. EsxA is routinely used to evaluate mycobacterial virulence in the laboratory and as a biomarker for tuberculosis in humans. The sequence of EsxA hinders deeper MS analysis beyond routine detection. Here we engineer the sequence of EsxA to add desirable tryptic properties aimed at improving complex MS analysis. We demonstrate that EsxA variants are amenable to MS analysis and remain functional in established *in vitro* and *ex vivo* assays of Esx-1-function. We provide the first demonstration of molecular engineering to specifically improve MS analysis of individual microbial proteins.

Proteomic detection using MS analysis is a standard approach to identify and quantify proteins. Protein mixtures are digested into peptide fragments using proteases. The most frequently used protease in MS analysis is trypsin, which cleaves following lysine (K) or arginine (R) with high fidelity. The size of the cleaved products, generally between eleven and 30 amino acids in length (Fig. 1), increases the likelihood of identification using MS and MS/MS methods. Due to the abundance of K and R in proteomes, most proteins when digested with trypsin produce peptides within this range that are sufficient for identification and for further quantitative analysis by MS (Fig. 1). However, MS analysis of some proteins is complicated by the amino acid composition; one is limited to the protease sites nature designed. In general, peptides ≤ 5 amino acids or greater than 30 amino acids perform poorly by chromatography or tandem MS and are not readily identified by common proteomics methods^1^,^2^. This subset of peptides, which comprises 28.1% of peptides generated by trypsin *in silico* for *M. tuberculosis*, are not commonly identified in experimental data and constitute a “dark/inaccessible proteotypic proteome” (Fig. 1). The dark-regions of the proteotypic proteome do not generally impact protein identification. Inconsistent recovery and poor reproducibility limit the quantitative analysis of specific peptides such as protein N- or C-termini, post-translational modifications, and domains with high or low distributions of K and R residues (Supplemental Fig. 1). Digestion with alternate proteases or using top/middle-down proteomics methods can be used to span dark regions^1^,^3^,^4^. While both of these techniques are promising, they can be less sensitive and more challenging than trypsin-based proteomics. The dark-regions of the proteome are also poor choices for MRM/SRM-based detection & quantification^5^. Some of the criteria for quantitative exclusion include the lack of a complete trypsin fragment, >20aa in length, and low digestion efficiency. Like trypsin, alternative proteases have inaccessible regions, necessitating larger sample size and multiple analyses to span a proteome. Finally, small proteins in particular are considerably constrained in the distribution of peptides due to a rarity of appropriate cleavage sites.

Although large MS peptides allow protein identification, stoichiometric quantification remains challenging. An underappreciated aspect of large peptides, which complicates MS analysis, is the relatively large number of sites capable of being modified in a non-physiological manner. Moreover, the number of sites that can result in a missed enzymatic cleavage is also large. Missed cleavages and non-physiological modification of peptides may only have a modest impact on peptide detection. However, they have a significant impact on quantification. Specifically, the large peptide is likely to exist in many proteoforms. No clean or consistent peptide form is observed across biological samples. Consequently, the signal for peptide quantification is diluted, creating severe challenges to peak integration. A typical MRM is a canonical cleavage peptide (high digestion efficiency and reproducibility), >7 and <20 amino acids in length with no M and typically avoiding C when feasible^5^.

Tuberculosis (TB) is an epidemic with a disease burden of ~9.6 million infections and 1.5 million deaths annually^6^. The Esx-1-exporter is a protein transport system which promotes the extracellular localization of mycobacterial virulence factors, including EsxA. EsxA is a major human T-cell antigen and a substrate of Esx-1^7^,^8^,^9^. Loss of Esx-1 is the chief cause of attenuation in the TB vaccine strain, *Mycobacterium bovis* BCG^10^,^11^,^12^. EsxA is used in blood-based diagnostics for TB including QuantiFERON TB Gold (Cellestis, Carnegie, Australia) and T-SPOT.*TB* (Oxford Immunotec, Oxford, UK) tests^13^. EsxA is also a central candidate included in the rational design of novel TB vaccines^14^,^15^. In the laboratory, EsxA secretion and activity are regularly used as reporters of Esx-1-function and mycobacterial virulence.

EsxA and its binding partner EsxB are produced in the cytoplasm of mycobacterial cells and transported across the cytoplasmic membrane by the Esx-1-exporter^8^,^9^,^10^. During *in vitro* growth, the EsxB and EsxA proteins are displayed on the mycobacterial surface and released or secreted into the culture supernatant via unknown mechanisms^14^,^15^. In *ex vivo* cellular infection models the Esx-1-system perforates the phagosomal membrane promoting interaction between the mycobacterial cell and the cytosol of the macrophage and modulation of the host innate immune response. Mycobacterial strains deficient for Esx-1-export are retained in the phagosome and are attenuated in *ex vivo* and *in vivo* infection models^16^,^17^,^18^,^19^,^20^.

Generally Esx-1-mediated protein secretion into culture medium during bacteriological growth correlates with virulence in *ex vivo* and *in vivo* infection models^10^. EsxA and other Esx-associated proteins are N-terminally acetylated^21^,^22^,^23^,^24^,^25^,^26^,^27^. How modification of the protein N-termini of Esx-associated proteins impacts mycobacterial virulence remains unknown. We have developed several MS approaches to measure the levels of EsxA on the mycobacterial cell surface and the modification of its N-terminus by acetylation^14^,^21^,^22^,^23^. However, routine use of these assays is complicated by the sequence of EsxA, which results in a large N-terminal tryptic fragment of 33 amino acids (Fig. 2).

The N-terminal tryptic fragment of EsxA has most of the criteria of a weak proteotypic peptide, including poor chromatographic reproducibility, further reducing accurate quantification and sensitive detection of EsxA between strains^5^. The substantially poor qualities of the EsxA N-terminus are well documented in peptide-libraries that catalog protein and peptide identification. The Peptide Atlas contains 13 proteoforms of the EsxA N-terminal peptide alone (Supplemental Table 1)^28^,^29^. A single modification of the N-terminus and monitoring for the loss or acetylation of the initiator Met residue means no fewer than 52 N-terminal peptides might need to be simultaneously monitored just to measure changes in the canonical N-terminus. These properties are reflected in the observed pool of N-terminal peptide fragments and the PeptideAtlas entry for EsxA which has suitability and observability scores ~10-fold less than internal peptides. This means that under typical conditions, your likelihood of identifying EsxA is high however your reproducibility, recovery and quantification of the N-terminus itself will be poor (Supplemental Table 1). Other proteases such as GluC generate size appropriate N-terminal peptide fragments of EsxA (Data not shown), but we find these proteases more costly and less reliable (complete and reproducible digestion) than trypsin.

Genetic manipulation of organisms to facilitate scientific inquiry is commonplace. Virtually all laboratory microorganisms are modified to circumvent natural barriers to study. Rational design to improve proteome analysis at the genetic level is a logical next step. However, genetic manipulation of the proteome has largely not been described. We sought to modify the sequence of EsxA at the chromosomal level to improve tryptic digestion and detection while retaining protein function.

## Results

### EsxA variants with improved MS performance are secreted from *Mycobacterium*

Prior studies have indicated that residues surrounding E12 are not required for EsxA secretion or functions that mediate virulence^30^. Moreover, changing residue E12 to alanine did not impact virulence of *M. tuberculosis* in a mouse model of infection^31^. We engineered the sequence of EsxA from *M. tuberculosis* to include an additional tryptic site following residue 12. We generated three variants which changed the E residue at position 12 to a K (E12K), an R (E12R) or a C (E12C) (Fig. 2). EsxA and EsxB are encoded by the *esxBA* operon and require each other for stability^8^,^32^. We introduced each mutation into an integrating plasmid containing the *esxBA* operon from *M. tuberculosis* (indicated with an “MT” subscript) and confirmed the mutagenesis by DNA sequencing analysis.

The Esx-1 exporter, as well as EsxA and EsxB, is highly conserved between *M. tuberculosis* and *Mycobacterium marinum,* a pathogenic mycobacterial species that is an established model for several aspects of *M. tuberculosis* pathogenicity and virulence^33^,^34^. One of the major benefits of using *M. marinum* as a model for *M. tuberculosis* is that this organism can be studied in a Biosafety Level 2 laboratory.

Deletion of the *esxBA* genes in *M. marinum* can be functionally complemented with the *esxBA* genes from *M. tuberculosis.* Therefore, we can study transport and function of the *M. tuberculosis* proteins in *M. marinum.* We introduced the plasmids expressing either wild-type or variant *esxBA* operons into *M. marinum* and *M. tuberculosis.* Because EsxA and EsxB are also required for Esx-1 function^8^,^9^, we expected that if the changes in the EsxA_*MT*_ sequence negatively impacted function, the EsxA_*MT*_ and EsxB_*MT*_ substrates would not be secreted into the culture medium. We prepared whole cell lysate and secreted protein fractions from both *M. tuberculosis* Erdman and *M. marinum* M strains expressing each EsxA_*MT*_ variant. We measured EsxB_*MT*_ production and secretion into the culture supernatant by both mycobacterial species using immunoblot analysis (Fig. 3). We detected EsxB_*MT*_ in the whole cell lysate and culture supernatant fractions from both wild-type *M. marinum* and wild-type *M. tuberculosis* strains but not in the fractions from the *M. marinum* Δ*esxBA* or *M. tuberculosis* Δ*esxA* strains (Fig. 3a). Expression of the wild-type or the three variant *esxBA*_*MT*_ operons restored EsxB_*MT*_ production and secretion from *M. marinum* and *M. tuberculosis*.

Because the changes to the EsxA_*MT*_ sequence disrupted the epitope recognized by the EsxA antibody used for immunoblot analysis, we measured the production and secretion of the EsxA_*MT*_ variants using proteomic analysis. We digested the protein fractions with trypsin and subjected the resulting peptides to LC/MS/MS analysis. Two novel tryptic peptides result from the introduction of a K or an R at the 12^th^ position at the N-terminus of EsxA (Fig. 2). To circumvent potential problems with tolerance of K and R, we also generated an E12C variant, which when chemically aminoethylated at the Cys residue, generates a tryptic substrate and the novel peptide M.TEQQWNFAGI[AEC-MAEC].

We readily detected the acetylated and unacetylated forms of the novel tryptic peptides (M.TEQQWNFAGIK. and M.TEQQWNFAGIR., respectively) corresponding to the first 12 amino acids of EsxA_*MT*_ in the whole cell lysate and culture supernatant fractions from *M. marinum* and *M. tuberculosis* strains expressing EsxA_*MT*_ E12K and EsxA_*MT*_ E12R (Fig. 3b,c and Supplemental Fig. 2). Following aminoethylation, we detected both the acetylated and unacetylated forms of the novel tryptic fragment corresponding to the EsxA_*MT*_ E12C protein in the culture supernatant from *M. tuberculosis* (Supplemental Fig. 3). From these data we conclude that the engineered EsxA_*MT*_ is facile for tryptic digestion and detection by LC/MS/MS analysis. Together, the immunoblot and the MS analyses demonstrate that the EsxA_*MT*_ variants are expressed and actively exported from *M. marinum* and *M. tuberculosis.* Moreover, the three EsxA variants can functionally promote secretion of EsxB_*MT*_ from both *M. marinum* and *M. tuberculosis.*

### EsxA variants retain function *in vitro* and *ex vivo*

We sought to further characterize the functionality of the EsxA_*MT*_ variants in *in vitro* and *ex vivo* Esx-1 assays. *M. marinum* lyses red blood cells in a contact-dependent, Esx-1-dependent manner^35^,^36^. As such, deleterious changes in EsxA_*MT*_ structure or function due to the E12C, E12K or E12R substitutions would result in a loss of hemolysis. As shown in Fig. 4a, wild-type *M. marinum* lysed sRBCs as indicated by an increased OD_405_ as compared to the negative PBS control. The Δ*esxBA* strain was non-hemolytic. Hemolytic activity was restored in *M. marinum* strains expressing either the wild-type or variant EsxA_*MT*_ proteins. These data demonstrate that the E12C, E12K and E12R variants of EsxA_*MT*_ can functionally promote hemolysis.

Infection of macrophages with virulent *M. marinum* at high multiplicity of infection (MOI) results in cytolysis of the host cell. Cytolysis requires perforation of the phagosomal membrane by Esx-1^19^,^20^. If the EsxA variants were nonfunctional, we expected decreased cytolysis of macrophages infected with strains expressing the EsxA_*MT*_ variants. We infected RAW 264.7 cells at an MOI of 5 and detected cytolysis by staining the macrophage monolayers 24 hours post infection using Calcein-AM and Ethidium Homodimer (Eth-D1). Calcein-AM is a membrane permeable dye that emits a green fluorescence when cleaved by cytosolic esterases in live cells. Eth-D1 is a nucleic acid stain that cannot cross intact cellular membranes. As shown in Fig. 4b,c, the infection of RAW 264.7 cells with wild-type *M. marinum* resulted in decreased viability (decreased Calcein-AM signal) and increased cytolysis (increased EthD-1 signal) as compared to the uninfected monolayer (mock). Infection with the Δ*esxBA* strain, which cannot access the cytosol, resulted in increased viability (robust Calcein-AM signal) and minimal cytolysis (decreased EthD-1 signal) as compared to infection with the wild-type strain. Expression of the wild-type or E12C, E12K or E12R variants of *esxBA*_*MT*_ in the Δ*esxBA* strain of *M. marinum* resulted in decreased viability and increased cytolysis as compared to the parent Δ*esxBA* strain. These data indicate that the EsxA_*MT*_ variants can promote Esx-1-mediated cytosolic access of *M. marinum* in an *ex vivo* macrophage infection model.

Phagosomal membrane perforation by the Esx-1-exporter triggers the cytosolic surveillance pathway (CSP) and the induction of IFN-β transcription in macrophages^17^,^19^,^20^,^30^. Therefore, induction of IFN-β transcript in macrophages infected by *M. marinum* or *M. tuberculosis* indicate that the Esx-1-exporter is functional. If EsxA_*MT*_ variants had altered activity, we expected a lack of the IFN-β transcript induction by strains expressing the EsxA_*MT*_ variants. We infected RAW 264.7 cells with *M. marinum* or *M. tuberculosis* strains expressing the wild-type or variant EsxA_*MT*_ proteins and measured the induction of IFN-β using qRT-PCR. Infection with either *M. marinum* or *M. tuberculosis* significantly induced transcription of IFN-β as compared to both the mock infected or Δ*esxBA* control strains (Fig. 4d, p = 0.0286). Expression of *esxBA*_*MT*_ in the Δ*esxBA* (*M. marinum*) or Δ*esxA* (*M. tuberculosis*) strains restored IFN-β transcription to levels at or above those induced by the wild-type strains. Expression of EsxA_*MT*_ E12C, E12K or E12R in the *esxBA* or *esxA* deficient strains also restored induction of IFNβ-transcription to levels at or above those induced by the wild-type strains. Together, these data demonstrate that the EsxA_*MT*_ variants promoted Esx-1-exporter activity in an *ex vivo* cell-based infection model.

## Discussion

Here, we provide the first example of the rational engineering of a gene of a microorganism to permanently improve proteomic detection while retaining measurable protein function. Our results demonstrate that the engineered changes to EsxA_*MT*_ that improved tryptic digestion and proteomic detection of the natural protein N-terminus resulted in functional EsxA protein in routine assays. Another engineering approach was used in *E. coli,* where trypsin sites were engineered into bacterial inner membrane proteins to facilitate the study of protein topology. However, these sites were not used to facilitate proteomic analysis^37^,^38^.

Other studies have suggested alternative protease combinations to address the lack of trypsin coverage^2^,^4^. While the use of protease combinations certainly has merit, our approach presented here is complementary. A strength of our approach is that altering the gene of interest prior to proteomic analysis will streamline the proteomic throughput, such that parallel digests are not required to span the protein of interest.

We envision several applications of our framework described here within *Mycobacterium.* The N-terminal acetylation of EsxA has been proposed to both impact the physical interaction between EsxA and EsxB and virulence^24^,^25^,^39^. The strains we have generated here could serve as parent strains for researchers interested in studying the biological significance of the N-terminus of EsxA and how its modification promotes mycobacterial virulence using proteomics approaches. Moreover, EsxA, and its binding partner, EsxB, have 20 paralogous proteins in *M. tuberculosis*^35^,^36^. In addition to the Esx-1-system, there are four additional type VII secretion systems, named Esx-2-5^40^. EsxN is a substrate of Esx5, and is required for virulence^21^. Esx-3 is required for metal homeostasis, and secretes EsxG and EsxH^41^,^42^. *In silico* analysis of the Esx-paralogs revealed that several of these proteins have regions that are unamenable to MS methods. In particular, many peptides generated from this population are outside of the ideal ranges for reproducible detection and are notably limited in the protein N-termini (Supplemental Fig. 4). The N-terminal peptides from EsxG, EsxC and EsxD have low suitability scores of 0.15, 0.1 and 0.1 respectively for their N-terminal peptide. The N-terminal peptides from EsxJ, EsxK, EsxM, and EsxP are too short to be observed (4 aa). The suitability scores of the N-terminal peptides from EsxO, EsxE and EsxF range between 0 and 0.01^28^,^29^. EsxH has a predicted N-terminal tryptic fragment of 67 amino acids which would be refractory to analysis and would be ideal for the approach outlined here. When just considering the Esx-like proteins, 40% of the N-termini alone are inaccessible. This places limitations on researchers’ ability to characterize changes in the processing or modification of the protein N-termini, which may be essential for disease^22^,^33^. In the *M. tuberculosis* proteome overall, we observed that 7.3% and 13.8% of peptides generated were greater than 30 amino acids for the empirical and *in silico* analyses respectively indicating that refractory proteins exceed the Esx-systems (Supplemental Fig. 1). As we demonstrate here, C and K substitutions introduce chemical side-chains which are readily modified to alter protease sensitivity, and may allow the quantification of otherwise intractable modified peptides.

**Figure 1:**
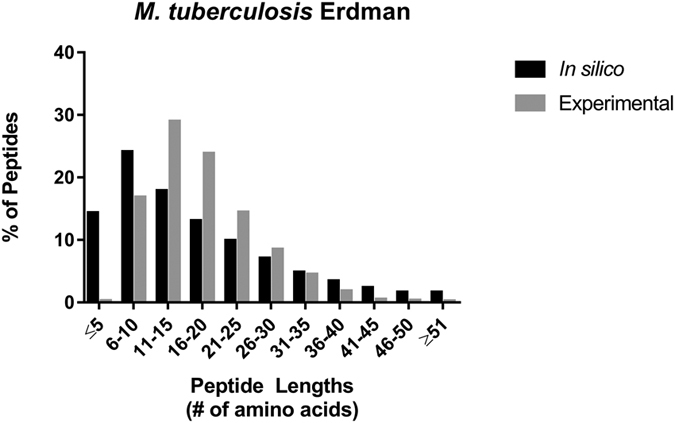
Experimental *vs. in silico* distribution of *M. tuberculosis* peptides produced from trypsin digestion. *In silico* and empirical length distribution of *M. tuberculosis* Erdman proteotypic peptides. *In silico* data are derived from a trypsin digested *M. tuberculosis* Erdman translated genome allowing one missed cleavage. High resolution nano-LC/MS/MS data for comparison were acquired from trypsin digested whole cell lysates of wild-type *M. tuberculosis* Erdman subjected to database search. Experimental analysis of peptide distributions were calculated from the peptide pool of the 1% FDR (7,659 peptides). Empirical data shows a bias against large peptides as compared to the translated genome and is overrepresented for peptides from 11–30 amino acids in length.

**Figure 2:**
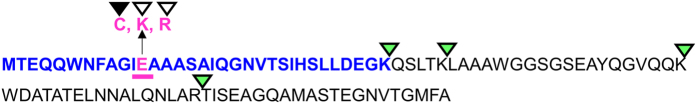
Molecular engineering of the EsxA protein to facilitate proteomic analysis. The amino acid sequence of EsxA from *M. tuberculosis.* Blue bolded residues indicate the natural N-terminal fragment generated by digestion with trypsin. Green triangles indicate other endogenous trypsin cleavage sites. The magenta underlined residue (E12) was changed to K or R, which introduces a novel site of trypsin cleavage (open triangle) or C residue which upon aminoethylation is a substrate for trypsin (closed triangle).

**Figure 3:**
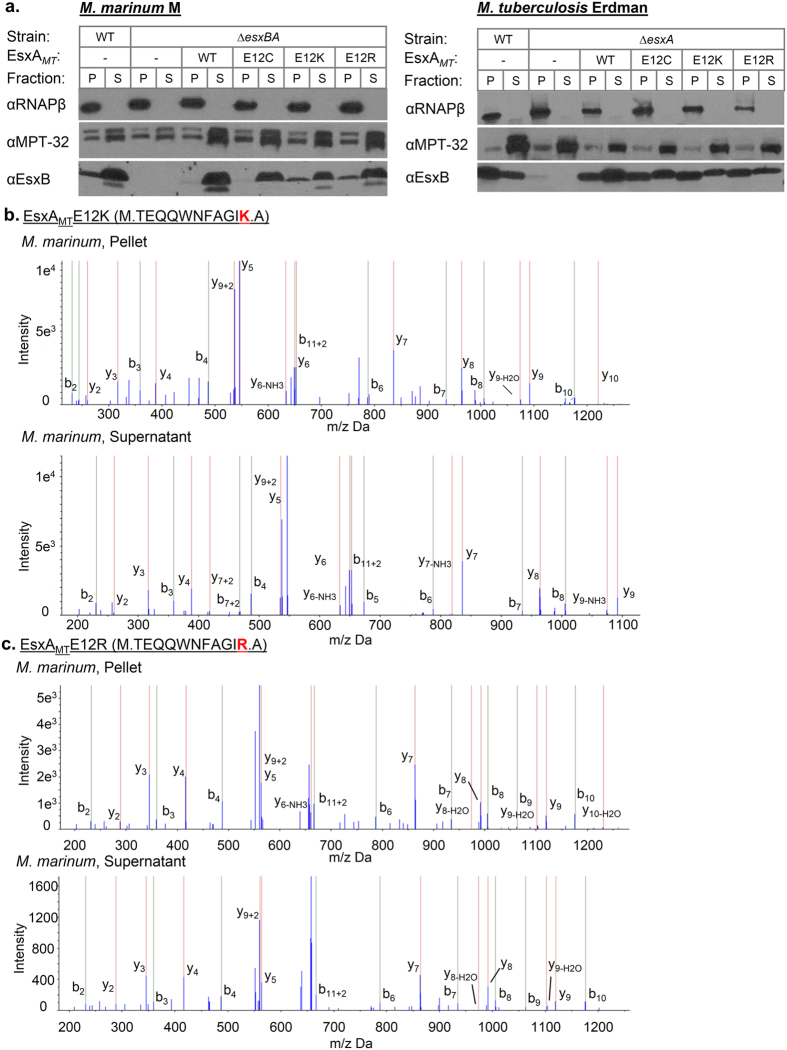
The engineered EsxA_*MT*_ variants are expressed and secreted from *Mycobacterium.* (**a**) Immunoblot analysis of protein fractions (pellet “P” and supernatant “S”) from *M. marinum* (upper) and *M. tuberculosis* (lower) demonstrating ESX-1-mediated secretion. RNAP-β subunit serves as a lysis control; MPT-32 serves as a control for Sec-mediated secretion and as a loading control. EsxB is an Esx-1-substrate and binding partner to EsxA. (**b**,**c**) Evidence of novel EsxA_*MT*_ tryptic peptides (K and R in (**b**,**c**), respectively) in pellet and supernatant fractions of *M. marinum* strains expressing EsxA_*MT*_ by LC/MS/MS analysis. Predominantly y and b-type ions are shown in the spectra for clarity.

**Figure 4:**
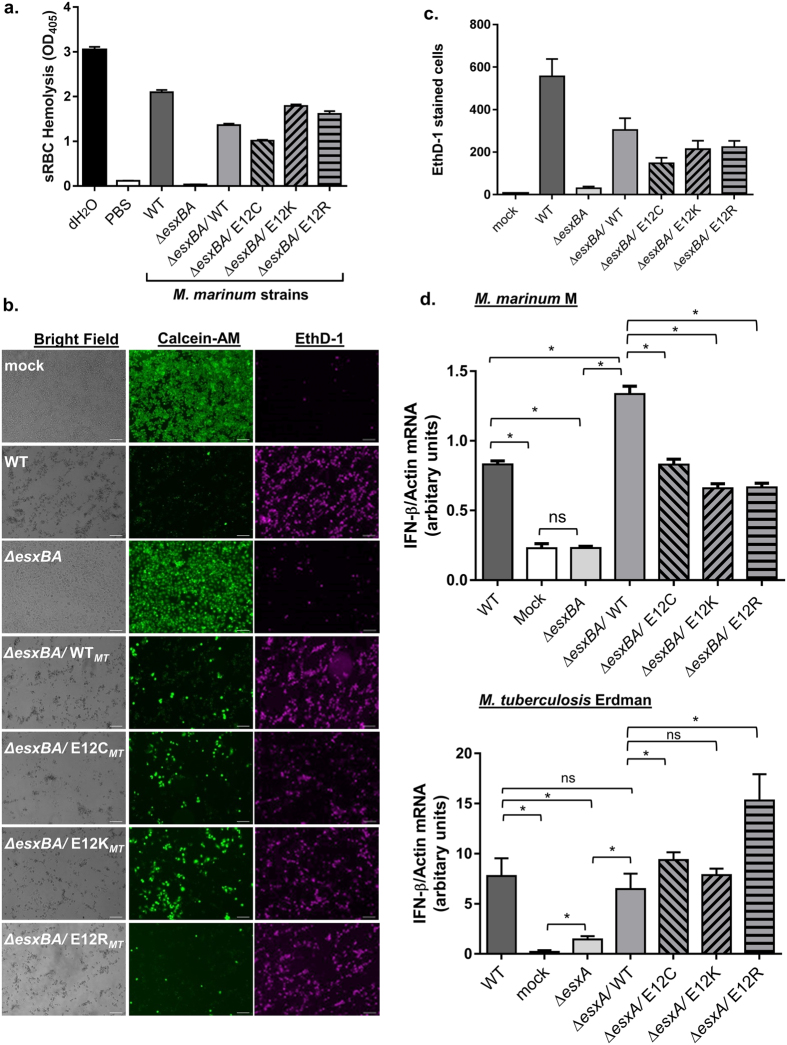
The engineered EsxA_*MT*_ variants expressed in *Mycobacterium* are functional *in vitro* and in an *ex vivo* infection model. (**a**) Hemolysis assays demonstrating the functionality of the Esx-1 system in *M. marinum*. Water serves as a positive control for sRBC lysis; PBS serves as a negative control for sRBC lysis. EsxA_*MT*_ variants expressed in each Δ*esxBA* strain follow the “/”. Hemolysis assays were performed on three biological replicates. Each replicate included three technical replicates. The error bars represent the standard deviation. The data shown is representative. (**b**) RAW 264.7 cells were infected with the indicated *M. marinum* strains at a multiplicity of infection (MOI) of 5 for 24 hours. Images were taken on Zeiss AxioObserver scope with 20X objective at 24 hpi. EthD-1 staining is representative of permeabilized cells. Calcein-AM stains live cells. Scale bar, 50 μm. (**c**) Cytolysis of the macrophage-like cells was measured by counting 10 independent fields of EthD-1 stained cells for each strain and then the counts were averaged. Error bars represent the standard deviation between the fields counted. We performed three independent biological replicates (each with two wells per strain) of the macrophage infections. For each biological replicate, we counted the permeabilized cells over ten fields per sample and reported the average. The error bars represent the standard deviation between the ten fields. The experiment shown is representative of the three infections. (**d**) qRT-PCR analysis demonstrating the induction of a type I IFN response by RAW 246.7 cells following infection by *M. marinum* (upper) or *M. tuberculosis* (lower) strains expressing each EsxA variant. IFN-β expression was normalized to actin expression as described previously. Data are representative of four biological replicates for *M. marinum* and two biological replicates for *M. tuberculosis.* Each biological replicate includes two technical replicates. Significance was determine using a Mann-Whitney U nonparametric test. *p = 0.0286. ns = not significant.

In cells, the natural distribution of K and R residues throughout the proteome limits complete coverage of the proteome or a specific set of proteins (Supplemental Fig. 1). Previous studies in *Saccharomyces cerevisiae* revealed that 56% of the theoretical peptides generated by trypsin are ≤6 amino acids, which are severely constrained for identification using proteomics^1^. In *M. tuberculosis,* at least 19.6% of the theoretical peptides generated by trypsin are ≤6 amino acids in length (Fig. 1 and Supplemental Fig. 1). Removal of existing K or R residues might be desired in cases where the ’natural’ polypeptide is too short for definitive MS assignment.

We envision that our approach would function with minor modifications for any target protein in any organism. Because this approach relies on altering single amino acids to introduce novel tryptic cleavage patterns, it is likely that the virtually all resulting protein variants can be designed to retain function. One consideration in applying this approach is that it has been reported that changing a single amino acid of a protein sequence could lead to the gain of new, undiscovered function (Reviewed recently by C.J. Jeffery^43^). It is formally possible that changing the 12th residue of EsxA to R, K or C could lead to new functional activity that we have not yet appreciated. However, here we demonstrate the canonical function of EsxA has not been lost.

Although we generated *esxA* variants using a PCR-based approach, a long-term implementation in higher organisms could be to employ other genome editing approaches including CRISPR to generate stable ‘proteome-ready’ lines-for research. Finally, we can envision an application by which several strains or cell lines, each bearing a unique C, K, or R substituted peptide in the same protein could serve as a proteomic ‘bar-coding’ enabling quantitative dissection of individual strains from mixed bacterial or complex cellular populations.

Overall, we provide a specific example of genetic manipulation of an *M. tuberculosis* virulence factor to improve proteomic analysis while retaining protein function. This approach is applicable to numerous targets across any systems to facilitate analysis of how alterations to proteins impact biological function.

## Methods

### Growth of mycobacterial strains

All mycobacterial strains used in this study are listed in Supplemental Table 2. All *Mycobacterium marinum* strains were derived from the M strain (ATCC BAA-535). *M. marinum* strains were grown at 30 °C and maintained in Middlebrook 7H9 defined liquid broth (Sigma-Aldrich, St. Louis, MO) with 0.5% glycerol and 0.1% Tween-80 (Fisher Scientific, Fair Lawn, NJ) or on Middlebrook 7H11 agar (Thermo Fisher, San Jose, CA) as previously described^26^. Kanamycin (20 μg/mL; IBI, Poesta, IA) or hygromycin (50 μg/mL; EMD Millipore, Billerica, MA) were added when appropriate. All *M. marinum* strains were grown and maintained in a BSL-2 laboratory under safety protocol (13–028), approved by the University of Notre Dame.

All *M. tuberculosis* strains were derived from *M. tuberculosis* Erdman (ATCC 35801), which was obtained from Jeffery S. Cox. *M. tuberculosis* strains were grown in 7H9 defined liquid broth with 10% Middlebrook OADC enrichment (Becton Dickinson and Company, Sparks, MD) and 0.05% Tween-80 at 37 °C. Kanamycin (20 μg/mL) or hygromycin (50 μg/mL) was added to the 7H9 broth when appropriate. All *M. tuberculosis* strains were grown and maintained in a BSL-3 laboratory under safety protocol (13-029) approved by the University of Notre Dame.

### Plasmid construction

The pMH406 plasmid (Kan^R^ gift of David Sherman, Hyg^R^ gift of Jeffery Cox) is an integrating plasmid bearing the *esxBA* genes from *M. tuberculosis* behind the mycobacterial optimal promoter (MOP)^7^,^42^. The pMH406 Hyg^R^ plasmid was the parent for the constructs expressed in *M. marinum.* The pMH406 Kan^R^ plasmid was the parent for the constructs expressed in *M. tuberculosis*.

Site directed mutagenesis of the pMH406 plasmid was performed exactly as described previously^44^. The *esxA*_*MT*_ E12C mutation was generated using OCR015 (5′-TTTCGCGGGTAT**CTG**CGCCGCGGCAAG-3′) and the reverse complement, OCR016, oligonucleotide primers where bolded residues indicate the altered codon. The *esxA*_*MT*_ E12K mutation was generated using the OCR011 (5′-TTTCGCGGGTAT**CAA**GGCCGCGGCAAG-3′) and the reverse complement, OCR012, oligonucleotide primers. The *esxA*_*MT*_ E12R was generated using the OCR013 (5′-TTTCGCGGGTAT**CAG**GGCCGCGGCAAG-3′) and the reverse complement, OCR014, oligonucleotide primers. All primers were purchased from Integrated DNA Technologies (IDT, Coralville, IA). The resulting mutagenized plasmids were confirmed by DNA sequencing analysis performed at the Genomics and Bioinformatics Core Facility at the University of Notre Dame using the MOPs forward (5′- TGCTTCCGGCTCGTATAATG-3′) and reverse (5′-GTCTTTCGACTGAGCCTTTC-3′) primers.

### Mycobacterial strain construction

Following confirmation by sequencing, the pMH406-based plasmids bearing either the wild-type or mutated alleles of *esxA*_*MT*_ were introduced by electroporation into the *ΔesxBA* or *ΔesxA* strains of *M. marinum* and *M. tuberculosis* strains, respectively, as described previously^45^. The resulting strains bearing the plasmids were selected on 7H11 agar containing 50 μg/ml hygromycin or 20 μg/ml kanamycin as required. Colonies were streak purified onto 7H11 agar with antibiotics and grown in 7H9 broth. Genomic DNA from each strain was purified as described previously^6^,^23^. Integration of the plasmid in each mycobacterial strain was confirmed by using the MOPs sequencing primers described above.

### Hemolysis assay

Sheep red blood cell (sRBC; defibrinated sheep red blood cells; BD, Sparks, MD) lysis assays were performed as previously described^44^ with minor modifications. *M. marinum* cells were washed three times with phosphate buffered saline (PBS) and then resuspended in 300 μl of PBS with 100 μl of sRBCs, collected by centrifugation, and incubated at 30 °C for 2 hours. For each strain, at least three biological replicates were performed. Each sample was read in triplicate. For all hemolysis assays, incubation of the sRBCs with dH_2_O served as a positive control for maximal lysis. Incubation of the sRBCs with PBS served as a negative control for lysis. Error bars are the standard deviations for triplicate readings. Data shown is from a single, representative assay.

### Cytotoxicity assays

The RAW 264.7 murine macrophage cell line (ATCC TIB-71) was maintained in DMEM (Life Technologies, San Diego, CA) supplemented with 10% heat inactivated fetal bovine serum (FBS) (HyClone Laboratories, Logan, UT) and maintained at 37 °C with 5% CO_2_, as described previously^14^,^22^. Cells were plated at 5 × 10^5^ cells per well in 24-well plates (Grenier Bio-one, Monroe, NC) for 24 hours and then infected with 2.5 × 10^6^ cells of *M. marinum.* Cells were incubated for 2 hours at 37 °C with 5% CO_2_ and then treated with 100 μg/mL gentamycin (Research Products International [RPI], Mount Prospect, IL) for 2 hours at 37 °C to kill external mycobacterial cells. The cells were then washed three times using PBS and fresh media was added. Staining of macrophages was performed using Ethidium homodimer-1 (EthD-1; Life Technologies, Eugene, OR) and the Live/Dead viability/cytotoxicity kit for mammalian cells (Life Technologies) at 24 hours post infection. The cells were imaged using an AxioObserver A1 inverted microscope (Zeiss, Thornwood, NY) as in Kennedy *et al.*^46^. We performed three independent biological replicates (each with two wells per strain) of the macrophage infections. For each biological replicate, we counted the permeabilized cells over ten fields per sample and reported the average. All magenta images were adjusted in the exact same manner to increase visibility (brightness +40% and contrast −40%).

### Macrophage infection and RNA extraction

The RAW 264.7 macrophage cell line was prepared and infected exactly as previously described^39^. RNA isolation was performed as described previously with the following minor changes: after the RNA binding step, the column was wash with 400 μL RNA wash buffer and the RNA was column digested by addition of 80 μL of the DNase I reaction mix (Zymo Research, Irvine, CA) directly to the column. After DNase I treatment, RNA was washed further according to the manufacturer’s instructions before being eluted. The RNA was quantified using a Nanodrop 2000 instrument (Thermo Fisher).

### qRT-PCR

Quantitative real-time PCR (qRT-PCR) was performed exactly as previously described^39^. The reaction and thermal cycling parameters were performed exactly as described in Mba Medie *et al.*^39^. The mRNA level of beta interferon (IFN-β) was normalized to that of actin. The IFN-β, and the β-actin primers oligonucleotide primers used here are: IFNβ-F (5′-CTGGAGCAGCTGAATGGAAAG-3′), IFNβ-R (5′-CTTGAAGTCCGCCCTGTAGGT-3′), β-actin-F (5′-AGGTGTGATGGTGGGAATGG-3′) and β-actin-R (5′-GCCTCGTCACCCACATAGGA-3′)^20^,^47^. We performed four biological replicates in *M. marinum* and two biological replicates in *M. tuberculosis*. Each biological replicate included two technical replicates. Statistical analysis was performed with GraphPad Prism, version 6.07 (GraphPad Software, San Diego, CA) using a Mann-Whitney U nonparametric test^48^.

### Esx-1 protein secretion assays

*M. marinum* protein secretion assays were performed exactly as described previously^26^,^45^. In short, *M. marinum* strains were grown in Middlebrook 7H9 broth and then diluted into Sauton’s liquid media with 0.01% Tween-80 at OD_600_ of 0.8 in 50 ml. Following 48 hours of growth, cell lysate and culture filtrate protein fractions were prepared and the protein concentration determined exactly as previously described^26^,^45^.

*M. tuberculosis* protein secretion assays were performed as previously described in Stanley *et al.* with minor changes^8^. Briefly, strains were cultured in Sauton’s liquid media supplemented with 0.05% Tween-80 and grown at 37 °C until OD_600_ 1–1.5. The cultures were subsequently diluted into Sauton’s liquid media to an OD_600_ of 0.05 with 0.005% Tween-80 and grown for 5–7 days at 37 °C, with gentle shaking. Cultures were harvested and fractions prepared as previously described^6^,^45^.

Protein fractions were run on Mini-Protean TGX Tris-HCl precast polyacrylamide gels (Bio-Rad, Hercules, CA) and transferred to a nitrocellulose membrane. Proteins were visualized using antibodies against EsxB (CFP-10) (1:5,000 [NR-13801; BEI Resources]), MPT-32 (1:5,000 [NR-13807; BEI Resources]), and RNA polymerase subunit β (RNAP-β) (1:5,000 [ab12087; Abcam] Abcam, Cambridge, UK) as previously described^39^.

### Proteomics/Mass Spectrometry

50 μg samples of cytosolic and secreted protein fractions were precipitated and digested as previously described^22^,^23^. Unless otherwise stated reagents were from Sigma-Aldrich (St Louis, MO). Briefly, acetone precipitated pellets were resuspended with vigorous vortexing in 50% 2,2,2 Trifluoroethanol, 50 mM ammonium bicarbonate, and Dithiotheritol (DTT) (Sigma, St Louis, MO) was added to a concentration of 10 mM. After incubation at 58 °C for 60 minutes, Iodoacetamide was added to a concentration of 20 mM and allowed to react in the dark for 15 minutes at room temperature. Additional DTT was added to quench the reaction. Aminoethylation was performed *in lieu* of alkylation using an identical procedure except the concentration of 2-Bromoethylamine HBr was increased to 50 mM × 2 1 hour reactions and ammonium bicarbonate was increased to 100 mM. The volume was brought to 200 μl in 50 mM ammonium bicarbonate and 500 ng of sequencing grade trypsin (Promega, Madison, WI) was added and incubated at 37 °C for 2 hours. An additional 500 ng of trypsin was added for overnight digestion. Digestion was quenched with addition of 5% formic acid (FA) (Thermo Fisher) until pH <4 and each sample was desalted with a C18 spin column using standard conditions (Protea, Morgantown, WV). Desalted-dried digests were resuspended to 500 ng/μl in 0.3% FA in water for LC/MS/MS analysis.

### LC/MS/MS

LC/MS/MS was performed essentially as described previously^22^,^23^. Briefly 2 μl samples (~1 μg digest) were injected onto a 100 μm × 100 mm C18BEH nano column with an Acquity nano UHPLC (Waters, Billerica, MA). A 90 minute gradient from 2–33% [A = H_2_O, 0.1%FA, B = Acetonitrile 0.1%FA (Burdick & Jackson, Morris Plains, NJ)] was run at a flow rate of 800 nl/min. MS & MS/MS were acquired on an Q-Exactive Orbitrap instrument (Thermo Fisher) running a TOP20 data dependent method^47^. MS and MS/MS spectra were converted to <mgf> peak lists using Proteome Discoverer 1.4 (Thermo Fisher) and exported for database searching. Database searching was performed using ProteinPilot/Paragon (ABSciex, Redwood City, CA) using a thorough-search parameter against a concatenated FASTA database of *M. marinum* M and *M. tuberculosis* Erdman strains respectively. This database contained a common contaminant file and the custom sequences of EsxA E12 C, K, and R substitutions to match the altered proteins. False discovery rates (FDR) were calculated using the method of Elias *et al.*^49^ and the instantaneous (Local) FDR from a quadratic fit was <10^−6^ % for EsxA peptides reported here. The 1% FDR population of proteins identified in culture filtrates and cytosolic fractions is typical for what we observe and is available for download through the MassIVE database http://massive.ucsd.edu with the accession # MSV000080101 or via ftp at ftp://MSV000080101@massive.ucsd.edu .

### Analysis of mycobacterial peptide distributions

For the data presented in Fig. 1, *in silico* digest of the *M. tuberculosis* Erdman strain proteome acquired from Uniprot (www.uniprot.org) was produced using the Pacific Northwest National Laboratories Protein Digestion Simulator (omics.pnl.gov) v2.2.5679; July 20, 2015. Digestion settings were set to full tryptic (KR not P) normal output with one missed cleavage allowed and a minimum fragment mass of 400 and maximum fragment mass of 6000 Da. The peptides generated were used to determine the distribution of peptide lengths. For the experimental data sets in-house digestion data of the *M. tuberculosis* Erdman strain was prepared using the filter–aided sample preparation method for LC/MS/MS analysis^50^. Briefly, 100 μg of cell pellet and culture filtrate fractions were denatured using 2.5% sodium dodecyl sulfate in 100 mM Tris, pH 8 with 100 mM DTT and reduced at 95 °C for 5 minutes. Samples were cooled and loaded onto 10 K microcon centrifugal filters (Millipore, Billerica, MA), volume added with 8 M Urea in 100 mM Tris (pH 8.0) spun and then alkylated with 50 mM iodoacetamide in 100 mM Tris and 8 M urea in the dark for 5 minutes. Four subsequent spin-washes were carried out using 8 M urea in 100 mM Tris pH 8.0. Two additional spin-washes in 100 mM ammonium bicarbonate were performed prior to digestion with trypsin at a 1:50 trypsin to substrate ratio at 37 °C for approximately 16 hours. Digests were spin-eluted and a second extraction with 500 mM NaCl was performed, followed by quenching with 5 μl FA. Desalting was performed using C18 spin columns (Protea, Morganstown, WV) and dried prior to LC/MS/MS analysis according to manufacturer’s instructions. Samples were re-suspended to 500 ng/μl and LC/MS/MS analysis was done essentially as described above. Identified peptides at a 1% FDR were used to determine peptide length distributions. Peptide lengths were determined by counting the number of amino acids. The percentage of peptides within each population was determined by taking the number of peptides within a bin and dividing by the total number of peptides at the 1% FDR. The percentage of peptides within each bin (≤5, 6–10, 11–15, etc.) were graphed for each individual data set (*in silico* and experimental). All calculations were done using Microsoft Excel. The missed cleavage rate for *in silico* digestion was chosen by analyzing the empirical data sets of the mycobacterial proteomes and determining the average number of missed cleavages per protein using the following calculation: (total number of missed cleavages/total number of peptides)*(average number of peptides per protein).

### Visualization of the *M. tuberculosis* Peptide Map

For the analysis in Supplemental Fig. 1, *in silico* digestion of the *M. tuberculosis* Erdman proteome was done as described above. Settings were set to zero missed cleavages allowed and no mass cutoff for the purpose of determining the entirety of the theoretical peptides generated from a perfect trypsin digestion. Peptides generated are representative of digestion of individual proteins from the N-to C-terminus (left to right). Peptide lengths were determined and used to create a heat map using the open source programming software, R^51^. For analysis of the Esx-like proteins in Supplemental Fig. 4, protein sequences were obtained from Mycobrowser^49^ and were analyzed *in silico* for digestion by trypsin with PeptideCutter (ExPASy). Protein graphics were generated using Microsoft Excel.

## Additional Information

**How to cite this article**: Reyna, C. *et al.* Rational engineering of a virulence gene from *Mycobacterium tuberculosis* facilitates proteomic analysis of a natural protein N-terminus. *Sci. Rep.* **6**, 33265; doi: 10.1038/srep33265 (2016).

## Supplementary Material

Supplementary Information
