# Supplementary material for: Rational engineering of a virulence gene from Mycobacterium tuberculosis facilitates proteomic analysis of a natural protein N-terminus
Source: Sci Rep. 2016 Sep 14;6:33265. doi: 10.1038/srep33265 (PMC5021934; doi:10.1038/srep33265)
Supplement: Supplementary Information [file srep33265-s1.pdf]

Supplementary Information

Rational engineering of a virulence gene from *Mycobacterium tuberculosis* facilitates proteomic analysis of a natural protein N-terminus.

Cristal Reyna, Felix Mba Medie, Matthew M. Champion*, and Patricia A. Champion*

Department of Biological Sciences, Department of Chemistry and Biochemistry, Eck Institute for Global Health, Center for Rare and Neglected Diseases, University of Notre Dame, Notre Dame IN

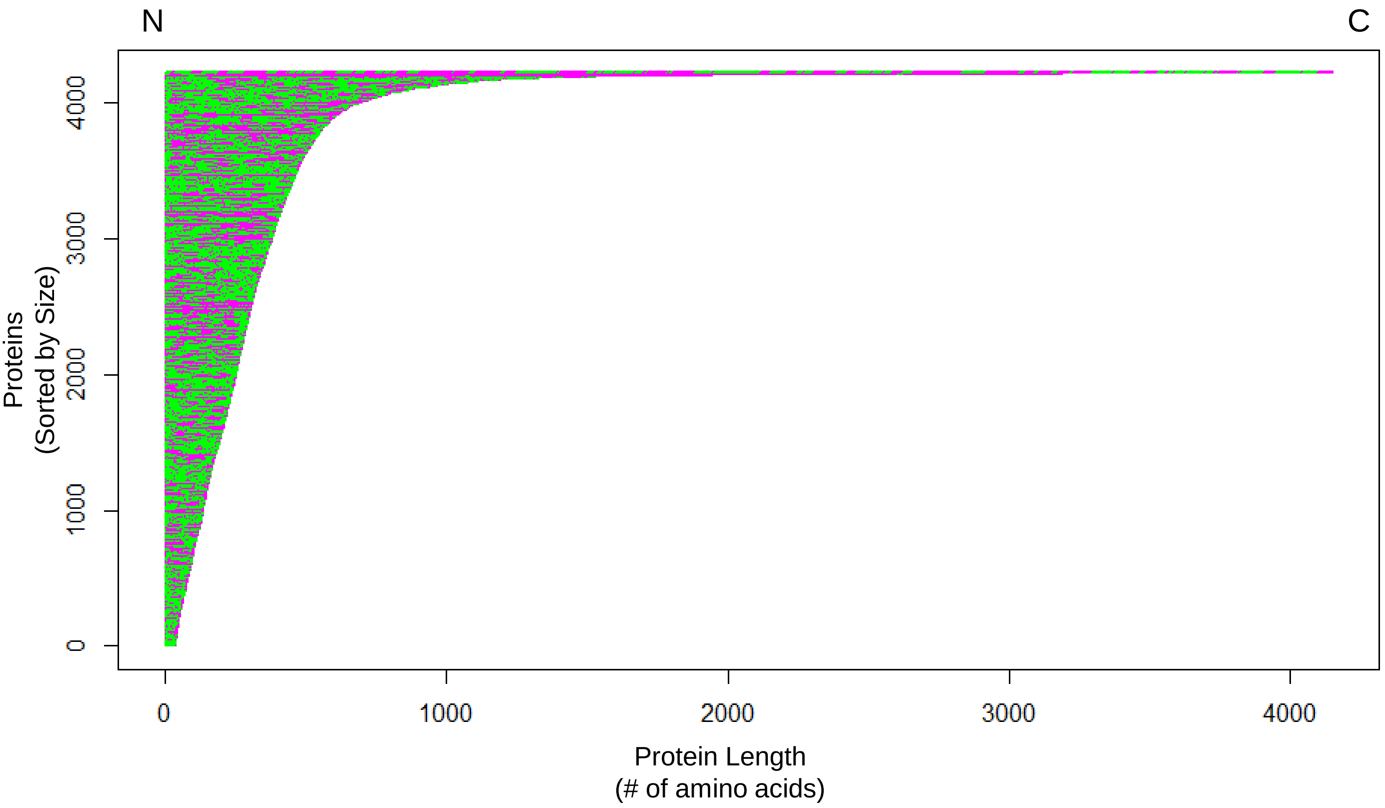

Supplementary Figure 1. Tryptic Peptide map of the complete *M. tuberculosis* proteome. Protein sequences of the *M. tuberculosis* Erdman proteome were acquired from UniProt and analyzed *in silico* for digestion by trypsin using the Pacific Northwest National Laboratories Protein Digestion Simulator. Each line indicates a protein from N to C terminus. Proteins were sorted by size. Magenta fragments are (≤5 amino acids) or (≥31 amino acids) in length; which are outside the bounds of the majority of identifications in bottom-up proteomics experiments. Green indicates fragments between 6-30 amino acids in length; which are typically recovered and have a higher likelihood of fragmentation and successful database-search.

a. EsxA$_{MT}$E12K (M.TEQQ[Dea]WNFAGI**K**.A)

M. tuberculosis, Pellet

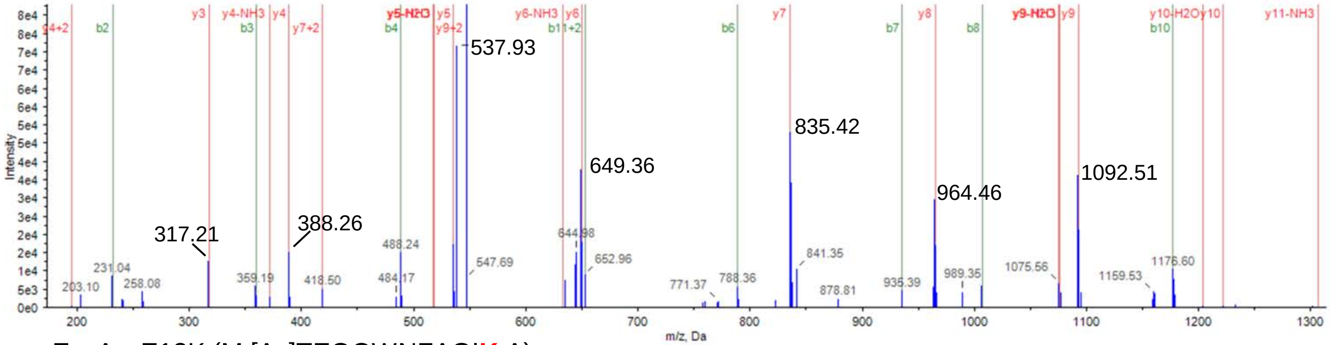

EsxA$_{MT}$E12K (M.[Ac]TEQQWNFAGI**K**.A)

M. tuberculosis, Supernatant

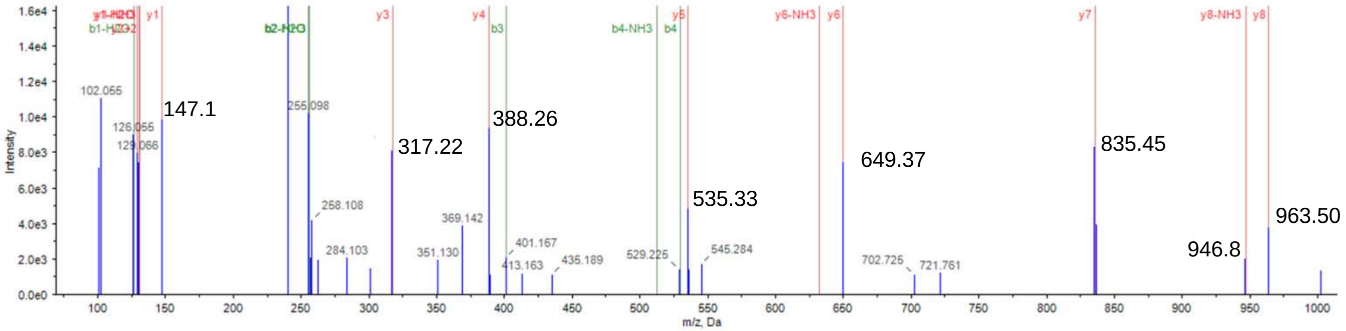

b. EsxA$_{MT}$E12R (M.TEQQWN[Dea]FAGI**R**.A)

M. tuberculosis, Pellet

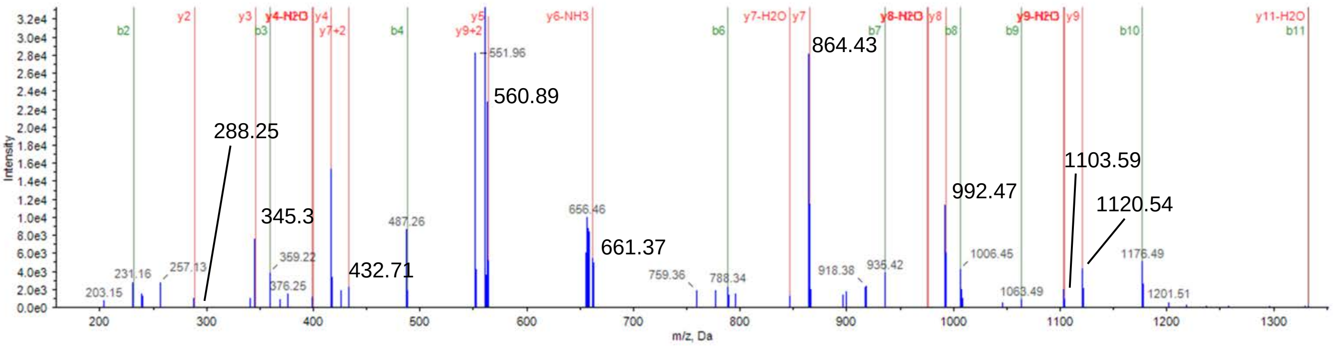

EsxA$_{MT}$E12R (M.TEQQWNFAGI**R**.A)

M. tuberculosis, Supernatant

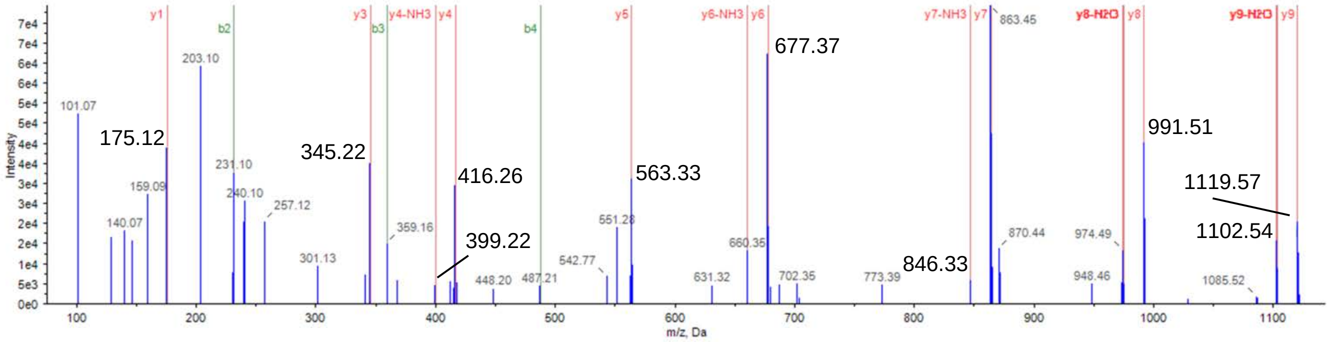

Supplementary Figure 2. The engineered EsxA$_{MT}$ variants are expressed and secreted from *M. tuberculosis.* **a**. Evidence of novel EsxA$_{MT}$ tryptic peptides (K and R in **a.** and **b.**, respectively) in pellet and supernatant fractions of *M. tuberculosis* strains expressing EsxA$_{MT}$ by LC/MS/MS analysis. TEQQWNFAGIK = 661.33 m/z $[M+2H]^{2+}$ TEQQ[Dea]WNFAGIK = 661.88 m/z $[M+2H]^{2+}$ TEQQWNFAGIR = 675.33 m/z $[M+2H]^{2+}$ TEQQWN[Dea]FAGIR = 675.88 m/z $[M+2H]^{2+}$. E12 K&R are likewise acetylated identically to WT strains as shown in the example spectrum in **a.**

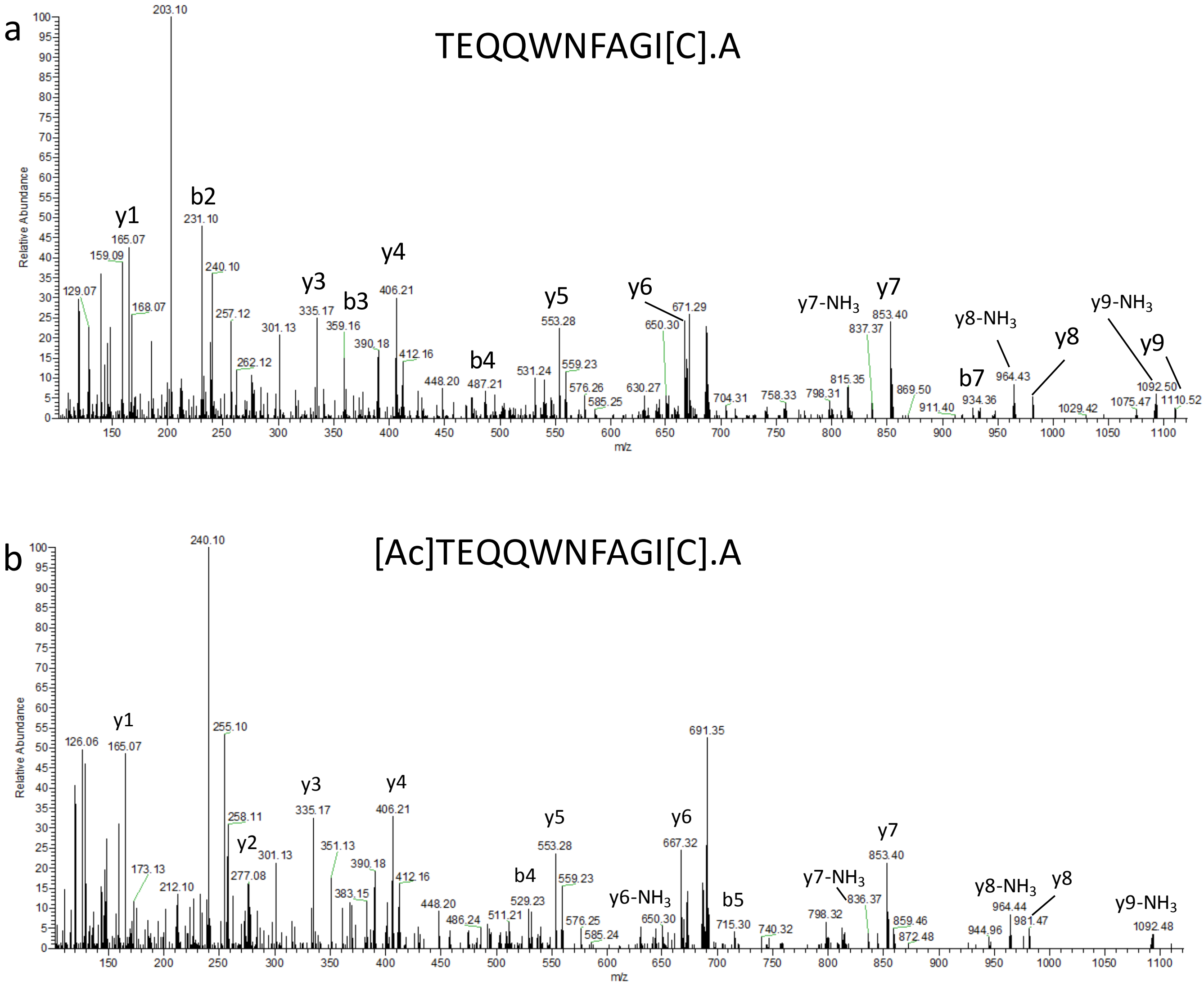

Supplementary Figure 3. **Annotated MS/MS spectra from aminoethyl-EsxA E12C**. Culture supernatants of *M. tuberculosis* expressing EsxA E12C were alkylated with 2-Bromoethylamine, digested with trypsin and LC/MS/MS was performed. Shown in **(a)** is the annotated MS/MS spectrum from the identified novel N-terminus of the protein and in **(b)** is the spectrum of the Nα acetylated proteoform of the N-terminus. (a.) m/z 670.31 $[M+2H]^{2+}$ (b.) m/z 691.31 $[M+2H]^{2+}$ Only y and b-type ions shown in both spectra for clarity.

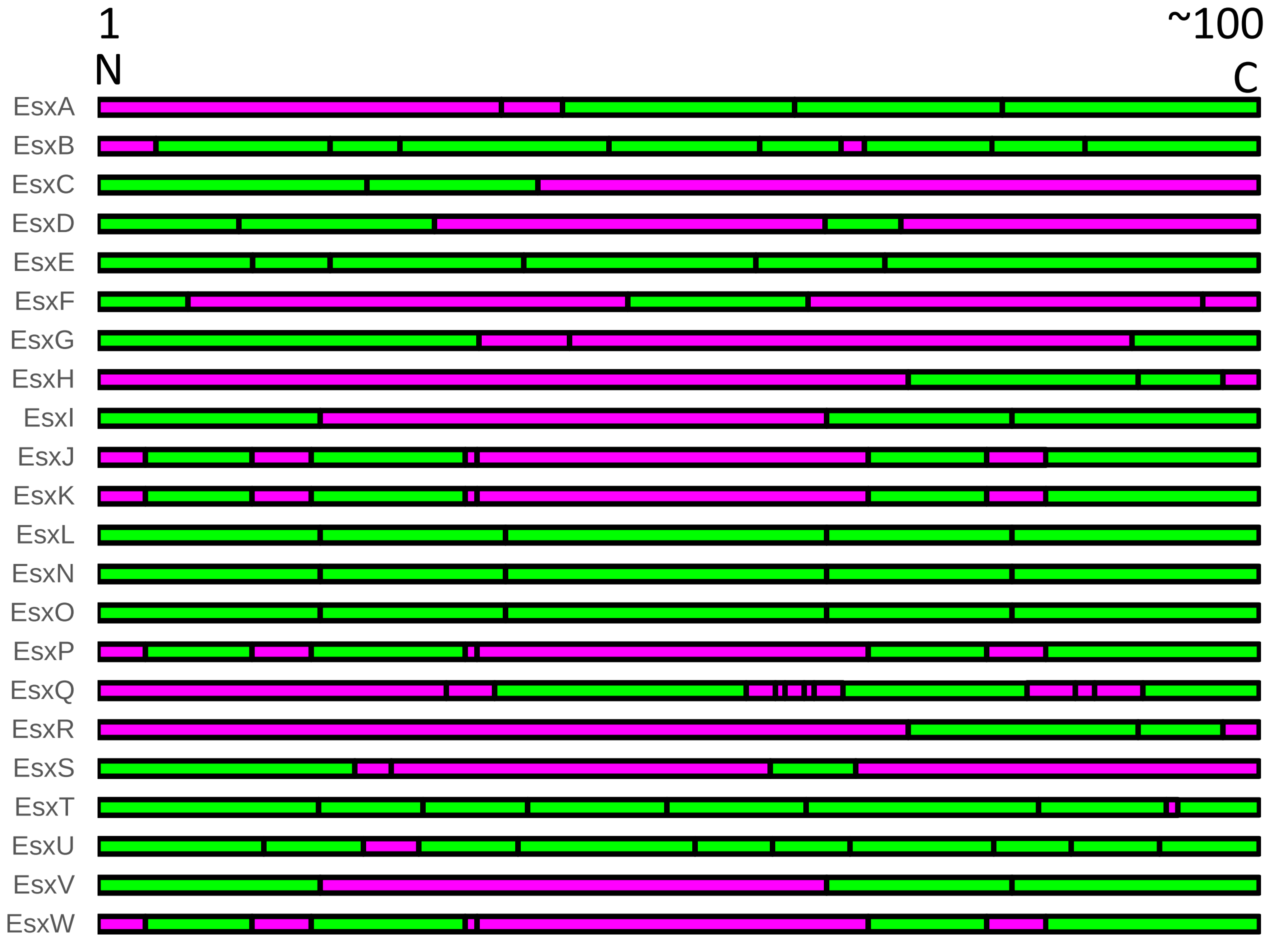

Supplementary Figure 4. **Peptide map of EsxA/EsxB paralogs in *M. tuberculosis.*** Protein sequences of the Esx proteins obtained from Mycobrowser were analyzed *in silico* for digestion by trypsin with PeptideCutter (ExPASy). Each line indicates a protein from N to C terminus. Magenta fragments peptide fragments ≤5 and ≥31 amino acids. Green fragments which are between 6-30 amino acids in length are more facile for MS and MS/MS analysis. EsxA/EsxB and its paralogs are small proteins and the limited tryptic fragments mean large portions of the N termini (9/22) and (13/22) of the protein interior are refractory to bottom-up MS/MS characterization.

Table S1: *Distinct Observed Peptides Table* From Peptide Atlas[a]

Accession	Pre AA	Sequence	Fol AA	ESS	Best Prob	N Obs	EOS	SSRT	N Prot Map	N Gen Loc	N Samples	N Subpep of
PAp01612785	K	LAAAWGGSGSEAYQGVQQK	W	1	1	714	0.89	24.7	1	1	8	0
PAp01613475	K	WDATATELNNALQNLAR	T	0.89	1	487	0.89	35.52	1	1	8	0
PAp01611962	R	TISEAGQAMASTEGNVTGMFA	-	0.69	1	220	0.67	33.8	1	1	6	0
PAp01616907	N	FAGIEAAASAIQGNVTSIHSLLDEGK	Q	0.10 [ntt]	1	9	0.44	42.23	1	1	4	3
PAp01616840	F	AGIEAAASAIQGNVTSIHSLLDEGK	Q	0.09 [ntt]	1	32	0.33	38.14	1	1	3	4
PAp01620262	R	TISEAGQAMASTEGN	V	0.09 [ntt]	1	16	0.33	17.06	1	1	3	3
PAp01626955	W	GGSGSEAYQGVQQK	W	0.09 [ntt]	1	13	0.33	9.55	1	1	3	5
PAp01627511	M	TEQQWNFAGIEAAASAIQGNVTSIHSLLDEGK	Q	0.09 [ntt]	1	12	0.33	45.06	1	1	3	0
PAp02152572	A	GIEAAASAIQGNVTSIHSLLDEGK	Q	0.09 [ntt]	1	10	0.33	37.62	1	1	3	5
PAp01624897	A	WGGSGSEAYQGVQQK	W	0.09 [ntt]	1	6	0.33	18.84	1	1	3	4
PAp02409406	A	SAIQGNVTSIHSLLDEGK	Q	0.08 [ntt]	1	14	0.22	32.36	1	1	2	9
PAp02409196	W	DATATELNNALQNLAR	T	0.08 [ntt]	1	8	0.22	29.32	1	1	2	1
PAp01628346	N	VTSIHSLLDEGK	Q	0.08 [ntt]	1	4	0.22	24.06	1	1	2	12
PAp01626951	A	AAWGGSGSEAYQGVQQK	W	0.08 [ntt]	1	3	0.22	20.06	1	1	2	2
PAp02413649	Q	GNVTSIHSLLDEGK	Q	0.08 [ntt]	1	3	0.22	27.96	1	1	2	11
PAp02154020	S	AIQGNVTSIHSLLDEGK	Q	0.08 [ntt]	1	2	0.22	30.79	1	1	2	10
PAp02409187	W	NFAGIEAAASAIQGNVTSIHSLLDEGK	Q	0.08 [ntt]	1	29	0.11	43.22	1	1	1	1
PAp01631060	R	TISEAGQAMASTEGNVTGM	F	0.08 [ntt]	1	5	0.11	26.3	1	1	1	1
PAp02412916	R	TISEAGQAMA	S	0.08 [ntt]	1	3	0.11	16.63	1	1	1	6
PAp02409232	K	LAAAWGGSGSEAY	Q	0.07 [ntt]	0.997	3	0.11	21.71	1	1	1	1
PAp02413464	E	AYQGVQQK	W	0.07 [ntt]	0.996	3	0.11	6.39	1	1	1	9
PAp01626916	G	GSGSEAYQGVQQK	W	0.07 [ntt]	0.996	2	0.11	9.32	1	1	1	6
PAp01626919	G	SEAYQGVQQK	W	0.07 [ntt]	0.997	2	0.11	8.8	1	1	1	7
PAp02413004	A	AASAIQGNVTSIHSLLDEGK	Q	0.07 [ntt]	0.994	2	0.11	33.1	1	1	1	6
PAp01612038	M	ASTEGNVTGMFA	-	0.07 [ntt]	0.998	1	0.11	22.56	1	1	1	1
PAp01615160	D	ATATELNNALQNLAR	T	0.07 [ntt]	0.999	1	0.11	28.66	1	1	1	2
PAp01620306	R	TISEAGQAMASTE	G	0.07 [ntt]	0.994	1	0.11	17.36	1	1	1	4
PAp02413732	S	EAYQGVQQK	W	0.07 [ntt]	0.995	1	0.11	7.72	1	1	1	8
PAp02465813	L	AAAWGGSGSEAYQGVQQK	W	0.07 [ntt]	1	1	0.11	20.51	1	1	1	1
PAp03008267	K	WDATATELNNALQN	L	0.07 [ntt]	0.996	1	0.11	29.42	1	1	1	1
PAp03008310	A	AWGGSGSEAYQGVQQK	W	0.07 [ntt]	1	1	0.11	19.23	1	1	1	3
PAp03008316	R	TISEAGQAMAS	T	0.07 [ntt]	0.997	1	0.11	15.38	1	1	1	5
PAp03008322	K	WDATATELNNALQ	N	0.07 [ntt]	1	1	0.11	30.1	1	1	1	2
PAp03008946	R	TISEAGQAMASTEGNVTG	M	0.07 [ntt]	1	1	0.11	21.24	1	1	1	2
PAp01625320	A	ASAIQGNVTSIHSLLDEGK	Q	0.06 [ntt]	0.972	1	0.11	32.44	1	1	1	7
PAp03008312	K	WDATATELNNA	L	0.06 [ntt]	0.985	1	0.11	22.61	1	1	1	3
PAp01855613	A	SAIQGNVTSIHSLLDEGKQSLTK	L	0.05 [mc,ntt]	1	2	0.11	34.87	1	1	1	1
PAp01854781	N	FAGIEAAASAIQGNVTSIHSLLDEGKQSLTK	L	0.05 [mc,ntt]	0.999	1	0.11	43.75	1	1	1	0

[a]. This is a table of the empirical observed peptides from EsxA deposited to the Peptide Atlas (Desiere, R,. *et al,.* 2006 and Deutsch, E., *et al.* 2010). Highlighted is the cannonical N-terminus which is infrequently and inconsistently recorded compared to the proteotypic (best) peptides from this protein. Its predicted Suitability Score (ESS) and Observability score (EOS) are substantially lower than highly observed peptides from the protein interior indicating poor usability as a peptide for routine study.

Table S1: Continued (Column Descriptions)

**Accession**	Peptide Atlas Identifier
**Pre AA**	Preceding (towards the N terminus) amino acid
**Sequence**	Amino acid sequence of detected pepide, including any mass modifications.
**Fol AA**	Following (towards the C terminus) amino acid
**ESS**	Empirical suitability score, derived from peptide probability, EOS, and the number of times observed. This is then adjusted sequence characteristics such as missed cleavage [MC] or semi-tryptic [ST], or multiple genome locations [MGL].
**Best Prob**	Highest PeptideProphet probability for this observed sequence
**N Obs**	Total number of observations in all modified forms and charge states
**EOS**	Empirical Observability Score, a measure of how many samples a particular peptide is seen in relative to other peptides from the same protein
**SSRT**	Sequence Specific Retention time provides a hydrophobicity measure for each peptide using the algorithm of Krohkin et al. Version 3.0 [more]
**N Prot Map**	Number of proteins in the reference database to which this peptide maps
**N Gen Loc**	Number of discrete genome locations which encode this amino acid sequence
**N Samples**	The number of samples in which this sequence was seen
**Subpep of**	Number of observed peptides of which this peptide is a subsequence

Table S2: Mycobacterial strains used in this study

Strain/Plasmid	Relevant Genotype	Source
***Mycobacterium marinum* M strain**	Parent strain	ATCC BAA-535/M
**Δ*esxBA***	M strain; deletion of *esxBA*; Kan^R Esx^-	[1]
**Δ*esxBA*/pMH406 *esxBA*$_{Mt}$**	Δ*esxBA* with *esxBA* behind the mycobacterial optimal promoter integrated at *attB*; Kan^R Hyg^R, Esx^+	This study
**Δ*esxBA*/pMH406 *esxBA*$_{Mt}$ E12C**	Δ*esxBA* with *esxBA* behind the mycobacterial optimal promoter integrated at *attB*; EsxAE12C mutation; Kan^R Hyg^R, Esx^+	This study
**Δ*esxBA*/pMH406 *esxBA*$_{Mt}$ E12K**	Δ*esxBA* with *esxBA* behind the mycobacterial optimal promoter integrated at *attB*; EsxAE12K mutation Kan^R Hyg^R, Esx^+	This study
**Δ*esxBA*/pMH406 *esxBA*$_{Mt}$ E12R**	Δ*esxBA* with *esxBA* behind the mycobacterial optimal promoter integrated at *attB*; EsxAE12R mutation, Kan^R Hyg^R, Esx^+	This study
***Mycobacterium tuberculosis* Erdman**	Parent strain	ATCC 35801, Gift of Jeffery Cox
**Δ*esxA***		[2]
**Δ*esxA*/pMH406 *esxBA*$_{Mt}$**	Δ*esxA* with *esxBA* behind the mycobacterial optimal promoter integrated at *attB*; Kan^R Hyg^R, Esx^+	This study
**Δ*esxA*/pMH406 *esxBA*$_{Mt}$ E12C**	Δ*esxA* with *esxBA* behind the mycobacterial optimal promoter integrated at *attB*; EsxA E12C mutation Kan^R Hyg^R, Esx^+	This study
**Δ*esxA*/pMH406 *esxBA*$_{Mt}$ E12K**	Δ*esxA* with *esxBA* behind the mycobacterial optimal promoter integrated at *attB*; EsxA E12K mutation Kan^R Hyg^R, Esx^+	This study
**Δ*esxA*/pMH406 *esxBA*$_{Mt}$ E12R**	Δ*esxA* with *esxBA* behind the mycobacterial optimal promoter integrated at *attB*; EsxA E12R mutation Kan^R Hyg^R, Esx^+	This study

1. Gao, L.Y. et al. A mycobacterial virulence gene cluster extending RD1 is required for cytolysis, bacterial spreading and ESAT-6 secretion. *Molecular microbiology* **53**, 1677-1693 (2004).
2. Stanley, S.A., Raghavan, S., Hwang, W.W. & Cox, J.S. Acute infection and macrophage subversion by *Mycobacterium tuberculosis* require a specialized secretion system. *Proceedings of the National Academy of Sciences of the United States of America* **100**, 13001-13006 (2003).
